# Construction and validation of a clinical prediction model for diabetic ketoacidosis

**DOI:** 10.3389/fmed.2025.1638927

**Published:** 2025-10-31

**Authors:** Chen Xu, Xingwen Jiang, Quanan He, Peng Xu

**Affiliations:** Department of Accident and Emergency, Yangtze River Shipping General Hospital, Wuhan, Hubei, China

**Keywords:** type II diabetes, diabetes ketoacidosis, multifactor logistic regression analysis, nomogram, dietary status, history of infection, HbA1c abnormality, hypokalaemia

## Abstract

**Background:**

Diabetes ketoacidosis (DKA) is a common and serious acute complication of diabetes mellitus. Globally, its incidence is on the rise, posing a serious threat to the life, health and quality of life of diabetic patients. In current clinical practice, although a variety of indicators are used to determine DKA, these indicators often have a lag and cannot effectively predict the occurrence of DKA at an early stage, resulting in some patients missing the best time for treatment.

**Objective:**

To investigate the risk factors for the development of DKA and establish a prediction model based on the information of type II diabetes mellitus.

**Methods:**

A total of 288 patients were collected in this study out of which 74 patients developed DKA. The patients enrolled in this study were randomly divided into a training set and a validation set according to a ratio of 7:3, with 201 patients in the training set and 87 patients in the validation set. The patients’ past medical history, dietary habits and relevant information during hospitalization were collected separately to study the correlation factors affecting the emergence of DKA in patients and to establish a prediction model.

**Results:**

Possibly relevant factors were included in a one-way logistic regression, and after analyzing the results: history of infection, dietary status, duration of diabetes mellitus longer than 3 years, history of smoking, history of alcohol consumption, abnormalities in liver function, abnormalities in HbA1c, and hypokalaemia were potential risk factors for the development of DKA, *P* < 0.2; The data obtained were further included in a multifactorial review: history of infection, dietary status (intemperate diet), duration of diabetes mellitus more than 3 years, HbA1c abnormality, and hypokalaemia were predictive factors for DKA (*P* < 0.05).

**Conclusion:**

This model provides a predictive tool for clinicians to identify high-risk patients with DKA at an early stage, which can help to take targeted preventive and intervention measures before the onset of the disease. However, the model was developed and internally validated using hospital-based data and that external validation is required before wider clinical application.

## 1 Introduction

### 1.1 Background and epidemiology of diabetes mellitus

Diabetes mellitus, a global chronic disease, is showing a high prevalence and high growth worldwide. According to the International Diabetes Federation, the number of people with diabetes worldwide reached 537 million in 2021 and is expected to increase to 783 million by 2045 (1–3).

### 1.2 Clinical significance of diabetic ketoacidosis

As one of the most serious acute complications of diabetes mellitus, diabetic ketoacidosis (DKA) is not to be underestimated (3–5). Domestically, studies have shown that the incidence of DKA among diabetic inpatients is about 10%–15%, while the incidence reported abroad varies from region to region and is about 4%–16% (6–8). DKA has a rapid onset and rapid progression, and once treatment is delayed, patients are very likely to fall into a coma or even face the risk of death, which undoubtedly significantly increases the chances of death of diabetic patients and the cost of medical care (9–12).

### 1.3 Problem statement of this study

Currently, scholars at home and abroad have conducted a lot of research on the pathogenesis, diagnosis and treatment of DKA. However, due to the complexity of the pathogenesis of DKA, which involves the intertwining of many factors, early diagnosis and prevention have always faced many difficulties, and most of the existing diagnostic methods rely on the detection of patients after the appearance of obvious symptoms, which makes it difficult to make an effective prediction of the disease in the early stage of its occurrence (11, 13–15). Although some foreign studies are committed to constructing prediction models, there is still room for improvement in the accuracy and universality of the models; domestic research in this field started relatively late, and further in-depth exploration is needed in the construction and validation of the models.

### 1.4 Research objectives

Therefore, it is urgent to construct an accurate and effective clinical prediction model for DKA. The aim of this study was to construct an accurate clinical prediction model for DKA by exploring in depth the risk factors associated with DKA and to validate the model rigorously. The construction of an accurate and effective clinical prediction model for DKA is of great significance for clinical prevention and treatment. On the one hand, it can help clinicians identify patients with high risk of DKA at an early stage, so that timely interventions can be taken to reduce the risk of DKA in diabetic patients; on the other hand, by intervening in advance, the prognosis of patients can be effectively improved, and the pain and medical burden of patients can be reduced, which can provide a powerful support for the health management of diabetic patients.

## 2 Materials and methods

### 2.1 Research object

This study was conducted on 288 patients with type II diabetes mellitus, 170 males and 108 females, who were admitted to our department from January 2022 to December 2023 for treatment. Ethics approval and consent to participate: We obtained a waiver of informed consent from the Ethics Committee. The Ethics Committee of Yangtze River Shipping General Hospital approved the study (Ethics approval number: WY-2023-032-12). This study adhered to the Declaration of Helsinki.

### 2.2 Research methods

This study was a retrospective case-control study. A total of 288 patients were collected, of which 74 patients developed DKA. Past medical history, dietary habits and information about the patients during hospitalization were collected separately to study the correlating factors affecting the development of DKA in the patients.

### 2.3 Collecting indicators

Gender, age, history of infection, dietary status, hypertension, hyperlipidaemia, duration of diabetes mellitus, history of smoking, history of alcohol consumption, abnormalities in liver function, abnormalities in renal function, abnormalities in HbA1c, hyponatraemia, and incidence of hypokalaemia were collected from the patients in this study.

### 2.4 Sampling strategy and sample size calculation

#### 2.4.1 Sampling strategy

In this study, we implemented a meticulously structured retrospective sampling approach to pinpoint patients with type 2 diabetes mellitus who were hospitalized between January 2022 and December 2023, utilizing the hospital’s electronic medical record system (Donghua HIS). We leveraged ICD-10 codes (particularly E11.6, which specifically indicates type 2 diabetes with ketoacidosis) in conjunction with clinical diagnostic criteria to effectively differentiate a case group, consisting of patients diagnosed with diabetic ketoacidosis (DKA), from a control group, made up of type 2 diabetes patients without a history of DKA. These two groups were then carefully matched in a 1:3 ratio.

#### 2.4.2 Sample size calculation

For the sample size calculation, we utilized the variable multiplication approach, which involves multiplying the number of predictor variables (in this case, 14) by a factor ranging from 10 to 20. This method yielded a minimum required sample size spanning from 140 to 280 cases. Ultimately, we included a total of 288 cases, thereby comfortably meeting the event count prerequisite essential for conducting a robust multiple regression analysis.

### 2.5 Inclusion and exclusion criteria

#### 2.5.1 Inclusion criteria

(i) Meets the diagnostic criteria for *Clinical guidelines for prevention and treatment of type 2 diabetes mellitus in the elderly in China (2022 edition)* (16);

(ii) Duration of the disease ≥ 1 year;

(iii) Age ≥ 18 years;

(iv) Patients in the study group presented with polyuria, irritable thirst and excessive drinking with nausea, vomiting, deep and fast breathing, and rotten-apple flavor in the mouth, as well as the appearance of a positive ketone body test in the blood with a pH < 7.35;

(v) Complete and faultless clinical data.

#### 2.5.2 Exclusion criteria

(i) Presence of history of surgery in the abdomen, gastrointestinal tumors and regular use of laxatives;

(ii) Those who have suffered from acute or chronic gastroenteritis or other malignant conditions;

(iii) Those who suffer from serious diseases of other systems, such as circulatory and urinary systems, or mental abnormalities;

(iv) Those currently taking anticholinergics, antispasmodics, and antidepressants;

(v) Those who are receiving other treatments or participating in other clinical studies.

### 2.6 Interpretation of some of the validation indicators mentioned in this study

(i) Infection history: clinical symptoms of local tissue or organ infection such as infection and fever were confirmed after the patient was admitted to the hospital.

(ii) Dietary status: including diabetic diet and unrestrained diet, mainly used to analyze patients’ dietary status and living status before hospitalization.

Diabetes diet: A standardized dietary regimen adhering to scientific principles for blood glucose control, emphasizing low-glycemic-index foods, quantified carbohydrate intake, and balanced combinations of high-fiber vegetables and lean protein. It strictly restricts added sugars and unhealthy fats while maintaining regular meal timing. The goal is to stabilize blood glucose variability, prevent acute complications (e.g., diabetic ketoacidosis), and fulfill individual nutritional requirements. Adjustments should be made dynamically based on the patient’s blood glucose levels, weight, and physical activity.

Unrestricted diet: This refers to eating habits that violate diabetes management goals. Patients may frequently consume high-sugar sweets, high-fat fried foods, and refined carbohydrates, accompanied by irregular meals, binge eating, or drinking on an empty stomach. This can cause blood sugar levels to fluctuate dramatically or remain uncontrolled for long periods of time.

(iii) Abnormal liver function: refers to the existence of cirrhosis and hepatitis in the patient’s past.

(iv) Abnormal renal function: patients with nephritis, nephrotic syndrome and other conditions in the past.

### 2.7 Statistical methods

SPSS 25.0 was used for data processing and statistical analysis in this study. Quantitative data conforming to normal distribution were expressed as mean ± standard deviation, and the differences between groups were analyzed by independent sample *t*-test. Comparisons between groups that did not follow a normal distribution were performed using non-parametric tests. Data for qualitative data were expressed as number of cases and percentage, and chi-square tests were used to determine whether there were differences between groups. Firstly, the patients were divided into the training set and validation set in a 7:3 ratio by randomization, and then analyzed based on the onset of DKA and various clinically relevant indicators in the training set. Potential risk factors for DKA were subsequently identified based on one-way logistic regression analysis of the collected data. For the univariate analysis, exposure factors with *P* ≤ 0.2 were selected and included in the multivariate analysis (17, 18). An independent risk factor for DKA was derived with DKA, and *P* < 0.05 was considered a statistically significant difference. Subsequent internal validation of the model further confirmed the reliability of the predictive model derived in this study.

## 3 Results

### 3.1 Table of values assigned to the relevant indicators in this study

See Table 1 for details.

**TABLE 1:** Table of values for relevant indicators.

Name	Variable assignment and description
Gender	Female-0, male-1
Infectious history	No-0, yes-1
Dietary status	Low sugar diet for diabetes-0, uncontrolled diet-1
Duration of diabetes	< 3 years-0, ≥ 3 years-1
Hepatic function abnormality	No-0, yes-1
Abnormal renal function	No-0, yes-1
state of stress	No-0, yes-1

### 3.2 Table of baseline characteristics of patients in both groups

This study randomly divided the patients included in this experimental study into a training set and a validation set in a ratio of 7:3. There were 201 patients in the training set and 87 patients in the validation set. The general information of the two groups of patients was included in the statistical study, *P* > 0.05. None of the differences in baseline characteristics between the two groups were statistically significant. See Table 2 for details.

**TABLE 2:** Comparison of baseline features between training and validation sets.

Variables	Total (*n* = 288)	Test (*n* = 87)	Train (*n* = 201)	Statistic	*P*
Age (year)	52.44 ± 4.76	51.92 ± 4.72	52.66 ± 4.77	*t* = −1.22	0.225
DKA, *n* (%)				χ^2^ = 0.97	0.325
0	214 (74.31)	68 (78.16)	146 (72.64)		
1	74 (25.69)	19 (21.84)	55 (27.36)		
Gender, *n* (%)				χ^2^ = 2.17	0.141
0	118 (40.97)	30 (34.48)	88 (43.78)		
1	170 (59.03)	57 (65.52)	113 (56.22)		
Infectious history, *n* (%)				χ^2^ = 0.30	0.586
0	192 (66.67)	60 (68.97)	132 (65.67)		
1	96 (33.33)	27 (31.03)	69 (34.33)		
Dietary status, *n* (%)				χ^2^ = 0.11	0.745
0	178 (61.81)	55 (63.22)	123 (61.19)		
1	110 (38.19)	32 (36.78)	78 (38.81)		
History of hypertension, *n* (%)				χ^2^ = 3.32	0.069
0	152 (52.78)	53 (60.92)	99 (49.25)		
1	136 (47.22)	34 (39.08)	102 (50.75)		
Hyperlipidaemia, *n* (%)				χ^2^ = 0.00	0.985
0	179 (62.15)	54 (62.07)	125 (62.19)		
1	109 (37.85)	33 (37.93)	76 (37.81)		
Duration of diabetes, *n* (%)				χ^2^ = 3.01	0.083
0	130 (45.14)	46 (52.87)	84 (41.79)		
1	158 (54.86)	41 (47.13)	117 (58.21)		
History of smoking, *n* (%)				χ^2^ = 0.06	0.814
0	152 (52.78)	45 (51.72)	107 (53.23)		
1	136 (47.22)	42 (48.28)	94 (46.77)		
History of alcohol consumption, *n* (%)				χ^2^ = 0.59	0.444
0	159 (55.21)	51 (58.62)	108 (53.73)		
1	129 (44.79)	36 (41.38)	93 (46.27)		
Hepatic function abnormality, *n* (%)				χ^2^ = 0.01	0.918
0	224 (77.78)	68 (78.16)	156 (77.61)		
1	64 (22.22)	19 (21.84)	45 (22.39)		
Abnormal kidney function, *n* (%)				χ^2^ = 0.14	0.705
0	190 (65.97)	56 (64.37)	134 (66.67)		
1	98 (34.03)	31 (35.63)	67 (33.33)		
HbA1c abnormalities, *n* (%)				χ^2^ = 0.55	0.457
0	142 (49.31)	40 (45.98)	102 (50.75)		
1	146 (50.69)	47 (54.02)	99 (49.25)		
Hyponatraemia, *n* (%)				χ^2^ = 3.54	0.060
0	254 (88.19)	72 (82.76)	182 (90.55)		
1	34 (11.81)	15 (17.24)	19 (9.45)		
Hypokalaemia, *n* (%)				χ^2^ = 0.00	0.997
0	245 (85.07)	74 (85.06)	171 (85.07)		
1	43 (14.93)	13 (14.94)	30 (14.93)		

### 3.3 One-way logistic regression analysis

The training set of 201 patients was included in the statistical analysis, of which 55 patients developed DKA. Possibly relevant factors were included in one-way logistic regression: history of infection, dietary status, duration of diabetes mellitus longer than 3 years, history of smoking (*P* < 0.096, OR = 1.70, 95% CI = 0.91–3.18), history of alcohol consumption (*P* < 0.001, OR = 3.30, 95% CI = 1.72–6.36), liver function abnormalities (*P* = 0.078, OR = 1.89, 95% CI = 0.93–3.82), HbA1c abnormalities, and hypokalaemia were potential risk factors for the development of DKA in patients with type II diabetes mellitus, *P* < 0.2. See Table 3 for details.

**TABLE 3:** One-factor logistic regression analysis based on training set.

Variables	β	SE	Z	*P*	OR (95% CI)
**Gender**					
0					1.00 (reference)
1	0.11	0.32	0.34	0.731	1.12 (0.60∼2.09)
**Infectious history**					
0					1.00 (reference)
1	1.52	0.34	4.54	< 0.001	4.58 (2.37∼8.85)
**Dietary status**					
0					1.00 (reference)
1	2.43	0.38	6.40	< 0.001	11.36 (5.40∼23.92)
**History of hypertension**					
0					1.00 (reference)
1	0.21	0.32	0.66	0.509	1.23 (0.66∼2.30)
**Hyperlipidaemia**					
0					1.00 (reference)
1	−0.09	0.33	−0.26	0.795	0.92 (0.48∼1.75)
**Duration of diabetes**					
0					1.00 (reference)
1	1.12	0.36	3.12	0.002	3.06 (1.52∼6.17)
**History of smoking**					
0					1.00 (reference)
1	0.53	0.32	1.67	0.096	1.70 (0.91∼3.18)
**History of alcohol consumption**					
0					1.00 (reference)
1	1.20	0.33	3.58	< 0.001	3.30 (1.72∼6.36)
**Hepatic function abnormality**					
0					1.00 (reference)
1	0.63	0.36	1.76	0.078	1.89 (0.93∼3.82)
**Abnormal kidney function**					
0					1.00 (reference)
1	0.29	0.33	0.89	0.372	1.34 (0.70∼2.56)
**HbA1c abnormalities**					
0					1.00 (reference)
1	1.02	0.33	3.08	0.002	2.78 (1.45∼5.34)
**Hyponatraemia**					
0					1.00 (reference)
1	0.23	0.52	0.43	0.665	1.25 (0.45∼3.48)
**Hypokalaemia**					
0					1.00 (reference)
1	1.19	0.41	2.91	0.004	3.27 (1.47∼7.28)

### 3.4 Multifactor logistic regression analysis

Incorporate risk factors identified through univariate analysis into multivariate analysis: history of infection (*P* < 0.001, OR = 5.67, 95% CI = 2.34–13.78), dietary status (intemperate diet, *P* < 0.01, OR = 14.66, 95% CI = 5.89–36.51), duration of diabetes mellitus for more than 3 years (*P* = 0.006, OR = 3.45, 95% CI = 1.43–8.28), HbA1c abnormality (*P* = 0.047, OR = 2.08, 95 CI = 0.90–4.80), and hypokalaemia (*P* = 0.032, OR = 3.20, 95 CI = 1.10∼9.28) were the factors showing predictive associations with DKA. See Table 4 for details.

**TABLE 4:** Multifactor logistic regression analysis based on training set.

Variables	β	SE	Z	*P*	OR (95% CI)
**Infectious history**					
0					1.00 (reference)
1	1.74	0.45	3.84	< 0.001	5.67 (2.34∼13.78)
**Dietary status**					
0					1.00 (reference)
1	2.69	0.47	5.77	< 0.001	14.66 (5.89∼36.51)
**Duration of diabetes**					
0					1.00 (reference)
1	1.24	0.45	2.77	0.006	3.45 (1.43∼8.28)
**HbA1c abnormalities**					
0					1.00 (reference)
1	0.73	0.43	1.71	0.047	2.08 (0.90∼4.80)
**Hypokalaemia**					
0					1.00 (reference)
1	1.16	0.54	2.14	0.032	3.20 (1.10∼9.28)

### 3.5 Plotting of nomograms

**FIGURE 1:**
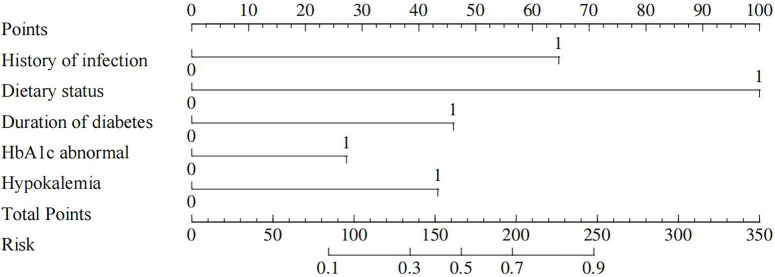
Nomogram predicts risk of DKA in type II diabetes patients.

**FIGURE 2:**
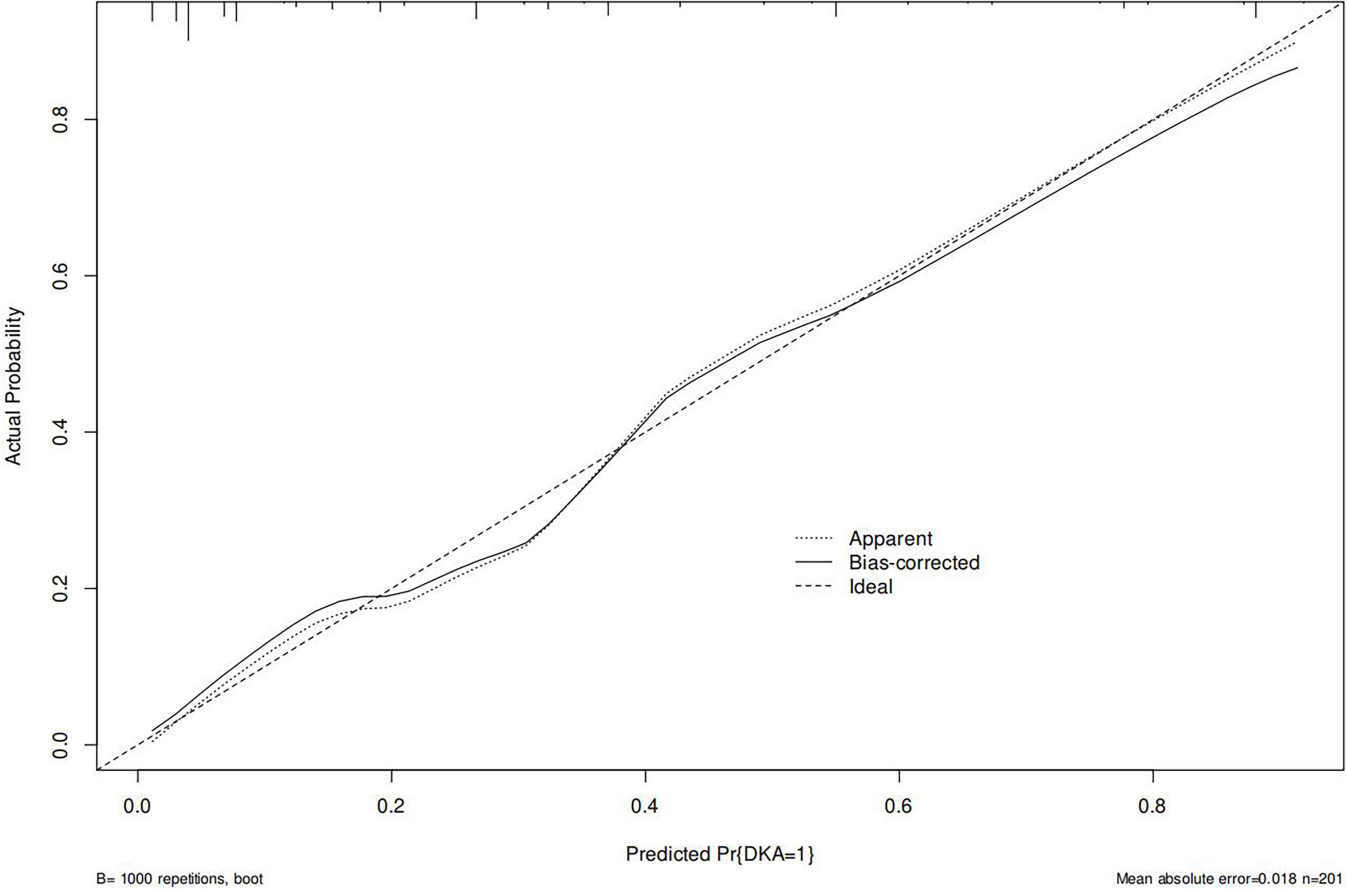
Internal validation of nomograms: calibration curves.

**FIGURE 3:**
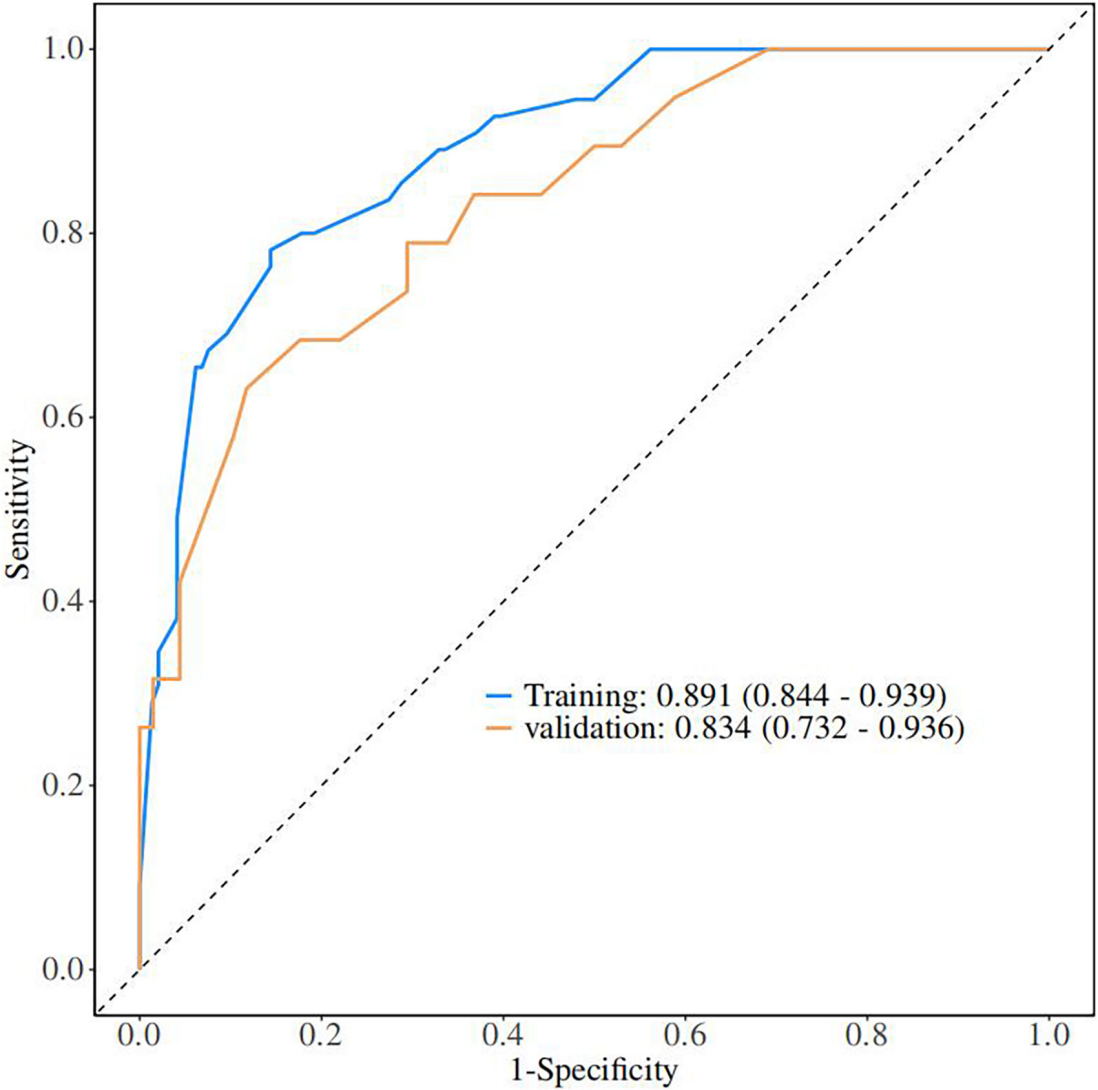
Validation of nomograms: ROC curves.

**FIGURE 4:**
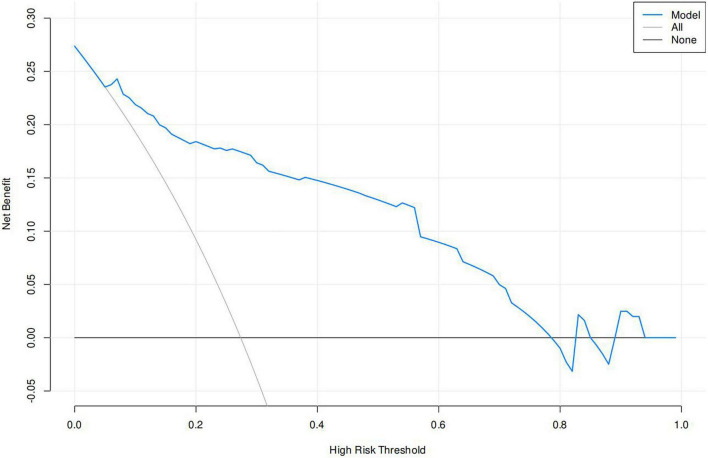
Decision curves in nomogram models.

A nomogram of the risk of developing DKA in patients with type II diabetes mellitus was constructed based on five predictive factors tested by multifactorial logistic regression analysis, see Figure 1 for details. Assign Nomo score to each independent risk factor. Sum up the total score based on the patient’s clinical characteristics, then locate on the total points axis. The value on the vertically downward corresponding Risk axis is the probability of DKA. The score of each independent predictor corresponds to the upper limit of the score of each independent predictor. The total score for each subject was the sum of each independent predictor score. The probability of developing diabetic ketoacidosis was determined by the total score on the risk axis for developing DKA in patients with type II diabetes. The model was subsequently validated internally: Internal validation of the nomograms was carried out using the Bootstrap method in R software with 1,000 repetitive samples. The calibration curve is close to the ideal curve, indicating that the nomogram predicts the incidence of DKA in type II diabetic patients with a high degree of agreement with the actual incidence, reflecting a certain predictive performance, see Figure 2 for details. The nomogram has a ROC curve for the training set with an AUC of 0.888 (95% CI = 0.846–0.929) and a ROC curve for the validation set with an AUC of 0.853 (95% CI = 0.780–0.925), see Figure 3 for details. It shows that the nomogram is a good discriminator of type II diabetic patients at high risk of developing DKA. The decision curve (DCA) for this nomogram shows that the model provides more net benefits than the all intervene or none intervene strategies when the threshold probability of an individual is greater than 0.05 in this column. This finding suggests that the nomogram model has a certain clinical application in predicting the occurrence of DKA in patients with type II diabetes mellitus, see Figure 4 for details.

## 4 Discussion

As a serious acute complication of diabetes mellitus, diabetic ketoacidosis (DKA) seriously affects patients’ health and quality of life, and has been a key area of concern in medical research at home and abroad. The construction of accurate and effective clinical prediction models for DKA is of great significance for early intervention and reduction of adverse outcomes. Some studies surface that patients’ age, type of diabetes, physiological and biochemical indicators such as blood glucose, blood ketone bodies, acid-base balance, electrolytes, clinical factors such as infections and insulin use, as well as lifestyle factors such as diet and stress, are closely related to DKA, and that genetic factors also have some influence (19).

### 4.1 An analysis of the results of clinical prediction models for DKA and the current situation at home and abroad

Two hundred and eighty-eight patients were included in this study, of whom 74 developed DKA. After univariate and multivariate analyses, a history of infection, dietary status without dietary moderation, duration of diabetes mellitus for more than 3 years, HbA1c abnormality, and hypokalaemia were determined to be the factors showing predictive associations with DKA (*P* < 0.05). Infection (*P* < 0.001, OR = 5.67, 95% CI = 2.34–13.78) is a common and important cause of DKA. An overseas study of adult DKA patients showed that about 40%–60% of DKA episodes were associated with infection (20, 21). Infection triggers a stress response in the body, prompting an increase in the secretion of catecholamines, cortisol and other glucagon, leading to a rapid rise in blood sugar; At the same time, metabolic disorders in the infected state accelerate lipolysis and increase free fatty acids, which in turn promotes ketogenesis and ultimately induces DKA (22, 23). In addition, other scholars have shown that respiratory and urinary tract infections are the most common types of infections in patients with DKA, and that poor infection control is strongly associated with recurrence of DKA (23, 24). Infection plays an important role in the pathogenesis of DKA, consistent with the results of the present study. Also, in terms of diet, there is growing interest in the association between binge eating and DKA. Overseas studies have found that irregular diets and diets high in sugar and fat can lead to large fluctuations in blood glucose, and long-term blood glucose instability can increase the risk of DKA. When patients consume too much high-sugar food, blood glucose rises rapidly, exceeding the regulatory capacity of pancreatic islet cells, insulin secretion is relatively or absolutely insufficient, lipolysis is intensified, and ketone bodies are produced in excess, thus triggering DKA (25, 26). There are also relevant reports in China that emphasize the importance of a reasonable diet for glycaemic control and prevention of DKA in diabetic patients, which further validates the rationality of dietary status (*P* < 0.01, OR = 14.66, 95% CI = 5.89–36.51) as an independent risk factor in this study (27). As the duration of diabetes increases, the patient’s insulin function declines. At the same time, studies have shown that patients with a disease duration of more than 3 years (*P* = 0.006, OR = 3.45, 95% CI = 1.43–8.28) have a significantly higher risk of developing DKA than those with a shorter disease duration (28–30). Prolonged hyperglycaemia has a toxic effect on pancreatic islet cells, causing a gradual decrease in their ability to secrete insulin, making glycaemic control more difficult, and making DKA more likely to occur in the event of stress factors, such as infections and poor diet. In addition, HbA1c (*P* = 0.047, OR = 2.08, 95 CI = 0.90–4.80) reflects the average blood glucose level over the past 2–3 months and is an important indicator for assessing long-term glycaemic control. A number of studies at home and abroad have confirmed that elevated HbA1c levels are positively associated with the risk of DKA (31, 32). An abnormal HbA1c means that the patient’s long-term glycaemic control is poor and metabolic disorders persist, increasing the likelihood of DKA. Finally, this study also confirms that hypokalaemia (*P* = 0.032, OR = 3.20, 95 CI = 1.10∼9.28) cannot be ignored in the pathogenesis of DKA. Insulin deficiency and hyperglycaemia result in the transfer of extracellular fluid potassium ions to the cells, while polyuria causes potassium loss, ultimately resulting in hypokalaemia. Hypokalaemia, in turn, further affects the effect of insulin action, exacerbates metabolic disorders and induces DKA (33–35).

### 4.2 Advantages of a clinical prediction model for DKA

The prediction model constructed in this study has unique advantages compared with the latest research literature at home and abroad. The AUC of the ROC curve for this nomogram was 0.888 (95% CI = 0.846–0.929). Some foreign studies have constructed models that incorporate more variables, but the models are complex and have limited clinical utility (36, 37). In contrast, the sample sizes of some domestic studies are small, and the stability and generalisability of the models are insufficient (38, 39). The predictive factors included in this study’s model were rigorously screened to ensure both model simplicity and good predictive efficacy. The probable reason for this is that the sample size of this study was moderate and covered diabetic patients with different characteristics, making the screening of risk factors more representative. In addition, the data were subjected to rigorous statistical analysis during the study to further optimize the model. In terms of intrinsic mechanisms, these predictive factors cover a number of key aspects such as infection, metabolism, diet, etc., which can comprehensively reflect the pathogenesis of DKA, thus improving the accuracy of model prediction.

### 4.3 Limitations

Although this study has achieved some results in the construction and validation of clinical prediction models for diabetic ketoacidosis (DKA), it inevitably has some limitations. Firstly, this is a single-center study with the sample originating from only one hospital. There are differences in genetic background, living habits, and medical conditions among populations in different regions, which may result in samples that are not fully representative of all diabetic populations, limiting the generalisability of the model when applied across different geographic regions. For example, some regions may have unique environmental factors or high prevalence of diseases that influence the pathogenesis of DKA, and a single-center sample that does not encompass these differences may reduce the predictive accuracy of the model in other regions. Secondly, this study only included common clinical factors such as history of infection, dietary status, and duration of diabetes, but did not consider the influence of emerging factors such as genes and gut flora on the development of DKA. Genetic polymorphisms may affect individual susceptibility to DKA, and different combinations of genes may result in different risks and courses of DKA when the organism is exposed to the same triggers. Gut flora are involved in the regulation of human metabolism, and their imbalance may interfere with glycaemic homeostasis and fat metabolism, which in turn may affect the development of DKA. By ignoring these factors, the model may omit key variables and fail to fully and accurately predict the risk of DKA onset. In addition, the study follow-up period was relatively short. The development of DKA may be dynamically affected by multiple factors such as long-term lifestyle and disease progression, which are difficult to capture in short-term follow-up and are not sufficient for the validation of long-term predictive efficacy of the model. In the long term, the patient’s physical condition, medication use, etc. may change, and the impact of these factors on the predictive accuracy of the model cannot be adequately assessed in a short period of time. Additionally, given that this study is retrospective in nature, despite the comprehensive variables collected and the fact that all variables were retrieved by researchers through the Donghua His medical record system, the study is inevitably subject to confounding bias and selection bias. Furthermore, retrospective studies are susceptible to reverse causality. This study primarily employed univariate and multivariate analyses to identify predictive factors for diabetic ketoacidosis (DKA), thereby constructing corresponding predictive models. And these methods represent the most fundamental approaches. Constrained by sample size limitations, we did not undertake more advanced statistical analyses such as propensity score weighting or competing risks models. The events-per-variable ratio may be low, raising the risk of overfitting. Although the AUC values for both the training and validation sets are relatively high, a certain discrepancy persists between them, making it impossible to completely rule out the possibility of overfitting. Additionally, calibration and decision curve results are mentioned qualitatively but not quantified. Furthermore, the predictive models lacked external validation sets to assess their generalisability, and non-linear methods such as Bayesian networks were not utilized to explore interactions between variables. Therefore, future studies should conduct prospective cohort studies with large sample sizes across multiple centers and perform Mendelian randomization studies using genetic variation as an instrumental variable to further exclude reverse causality.

### 4.4 Future direction and prospects

In the future, we plan to conduct a multi-center, large-sample study on the clinical prediction model of DKA, including diabetic patients from different regions and races, to comprehensively consider the effects of geographic and genetic differences on the onset of DKA, so as to improve the general applicability of the model. At the same time, the intrinsic links between genes, intestinal flora and other emerging factors and DKA will be explored in depth, and advanced gene detection technology and intestinal flora analysis will be used to clarify their specific roles in the pathogenesis of DKA, which will be incorporated into the prediction model to enhance the accuracy and comprehensiveness of the model. In addition, the follow-up time is greatly extended to closely track the long-term health status of patients, dynamically monitor the changes of various influencing factors, verify the long-term predictive effect of the model, and provide a more reliable predictive tool for the clinic, which will help in the early prevention and precise treatment of DKA.

## 5 Conclusion

Through the in-depth profiling in this study, we have successfully identified history of infection, dietary status without dietary moderation, duration of diabetes mellitus up to 3 years or more, HbA1c abnormality, and hypokalaemia as predictive factors for the development of DKA. Clinical prediction models constructed on the basis of these findings enable clinicians to identify patients at high risk of DKA early. In practice, doctors can use this model to develop targeted prevention and intervention strategies in advance, before the disease has even started. For example, focusing on strengthening the prevention and control of infection, paying close attention to the patient’s dietary status and giving reasonable dietary guidance, optimizing the blood glucose management plan according to the patient’s specific condition, and so on. Through these effective measures, it is expected to effectively reduce the incidence of DKA and the morbidity and mortality rate, and thus significantly improve the overall prognosis of diabetic patients, providing a more solid guarantee for the healthy life of patients.

## Data Availability

The raw data supporting the conclusions of this article will be made available by the authors, without undue reservation.
